# Francisco de Castro: Localizationism, intelligence and the frontal lobe

**DOI:** 10.1590/1980-57642016dn11-030013

**Published:** 2017

**Authors:** Pedro Sudbrack Oliveira, Eliasz Engelhardt, Marleide da Mota Gomes

**Affiliations:** 1 Fellow, Postgraduate Programs, Department of Internal Medicine, Medical School: Federal University of Rio de Janeiro, RJ, Brazil.; 2 Professor (retired), Deolindo Couto Institute of Neurology: Federal University of Rio de Janeiro, RJ, Brazil.; 3 Associate Professor, Deolindo Couto Institute of Neurology: Federal University of Rio de Janeiro, RJ, Brazil.

**Keywords:** Francisco de Castro, localizationism, cortical functions, frontal lobe, Francisco de Castro, localizacionismo, funções corticais, lobo frontal

## Abstract

This article addresses the largely unknown legacy of Francisco de Castro regarding the neurological sciences. His essay "Psychogenic Cortical Centers", written in 1881 for his admission to the Imperial Academy of Medicine in Rio de Janeiro, is a refined appreciation of the theory of localized cortical functions that was in evidence in Europe in the second half of the nineteenth century.

## RESUMO

O presente artigo apresenta o legado pouco conhecido de Franciso de Castro, referente às ciências neurológicas. Seu ensaio "Centros Corticaes Psychogenicos", escrito em 1881 como uma forma de ser admitido como membro na Academia Imperial de Medicina, no Rio de Janeiro, é uma refinada excursão pela teoria das funções corticais localizadas, em destaque na Europa na segunda metade do século XIX.

## INTRODUCTION

**Figure 1:**
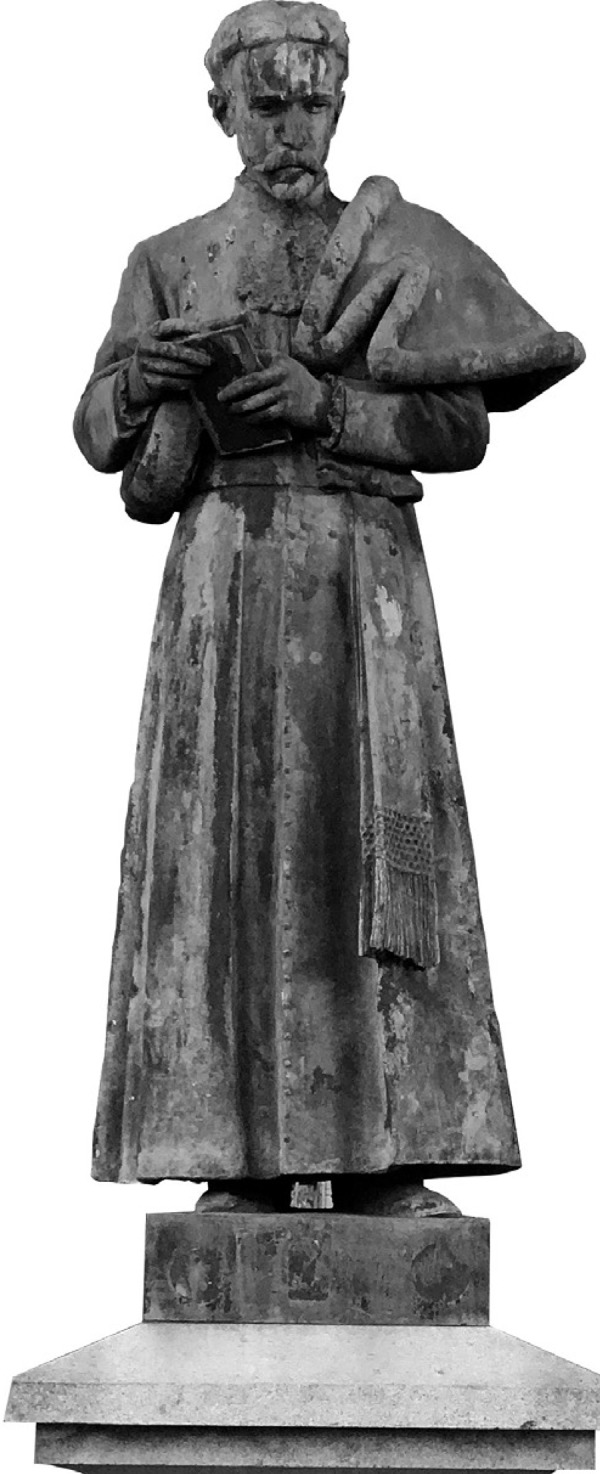
Francisco de Castro statue (1910) – The first raised to a physician, in Brazil, by the sculptor Bernardelli. Now, it is located at the Health Sciences Center, Federal University of Rio de Janeiro.

Francisco de Castro (1857-1901) (Figure 1) was a talented physician and an icon of Brazilian medicine in the late nineteenth and early twentieth centuries. In 1874, at age sixteen, he entered the Bahia Faculty of Medicine, the oldest Brazilian medical school.^1^ It was during those years that he befriended Guilherme de Castro Alves, brother of Antônio Frederico de Castro Alves, one of the greatest Brazilian poets. Both of them inspired Francisco de Castro's approach to the literature and had an influence in his poetry book "Wandering Harmonies" (entitled Harmonias Errantes in Portuguese), linked to the Romantic tradition. It received a preface written by Machado de Assis and was published in 1878.^2^

He finished his medical training in 1879, the same year he married Maria Joana Monteiro Pereira. Their son Aloysio de Castro grew to be a co-founder of Brazilian neurology.^1^ After finishing his studies, Castro assumed an academic position at Rio de Janeiro Faculty of Medicine. As a teacher, he was deeply admired by his colleagues and students.^3^ His book "Treaty on Clinical Propedeutics" published in 1896 was highly regarded by many, and, according to his son Aloysio de Castro, was responsible for the introduction of a rigorous clinical method in Brazilian medical schools.^1^ Besides, Carlos Chagas, an illustrious Brazilian researcher, acknowledged Castro's wisdom: "There is no science on the outside where there is no logic on the inside. That was the sentence that I once heard and that synthesized, in both practice and teaching, the exceptional virtues of his high clinical personality."^1^

## FORAY INTO NEUROSCIENCES: LOCALIZATIONIST THEORY

**Figure 2:**
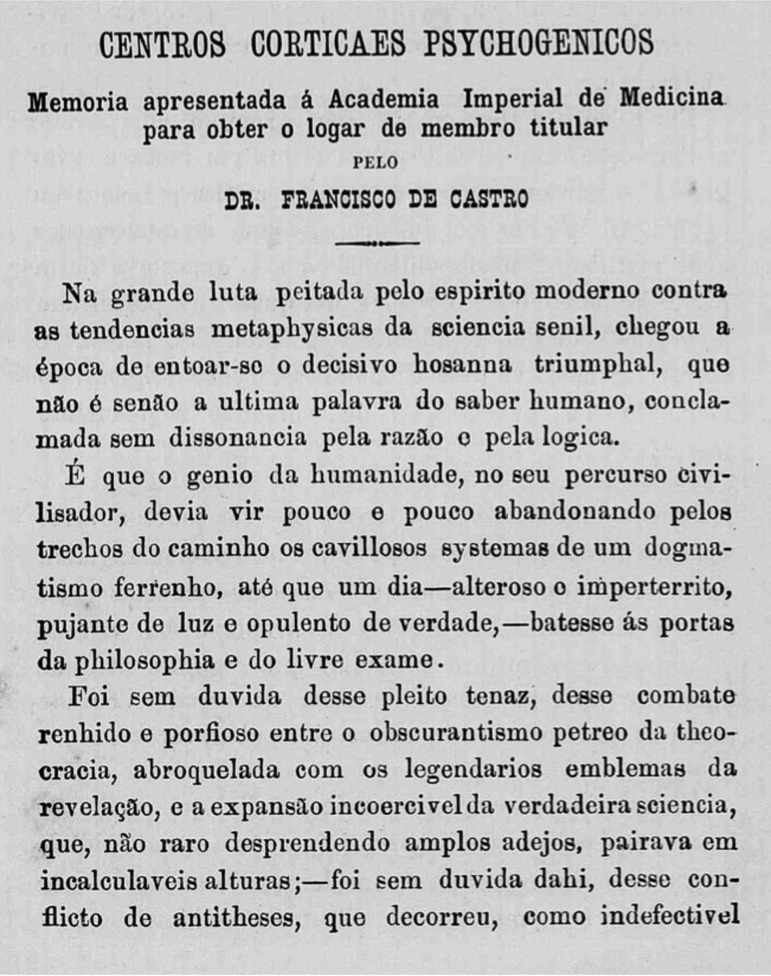
First page of Castro's "Psychogenic Cortical Centers".

Castro's interest in the nervous system can be acknowledged in his essay "Psychogenic Cortical Centers" (Figure 2). which he presented in 1881 to become a member of the Imperial Academy of Medicine, in Rio de Janeiro.^4^ It mainly dealt with the cortical localization of functions, a doctrine which grew in those days due to increasing evidence from animal experiments and *post mortem* analysis of pathological human brains. After an introduction full of philosophical inquiries and erudition, he initiates his essay on the localization theory: "The foundation of all intellectual acts and the most elementary psychological factors, *alma mater* of all ideas, is sensation. Having bordered the cortical territory where the phenomenon of sensation is held, in its last and most complex instance, we will have surrounded the focus of very active ideogenesis and established the capital piece of the process of localizations." He then brings evidence on the existence of cortical centers related to sensory functions and quotes David Ferrier (1843-1928), a Scottish neurologist and psychologist who had done experimental lesions and electrical stimulation of the cerebral cortex of numerous species, including primates.^5^^,^ 6 It is now known that Ferrier correctly identified several sensory "cortical centers", such as the auditory, olfactory and gustatory, providing topographical maps.^6^ A short time before the publication of Castro's essay, the linkage of vision to the occipital lobe was described by Hermann Munk (1839-1912), a German physiologist.^7^^,^ 8 However, Castro was apparently unaware of those studies.

In "Psychogenic Cortical Centers", Castro argued that sensory experiences physically change the brain, and in those sensory areas reside the most basic ideas that, through integration with motor centers, ultimately lead to complex behavior. Furthermore, Castro stands that those initial *energy of ideas* affects not only movement, but also influences other ideas, in a process culminating on intelligence.^4^ On this topic, he criticizes the assumption made by some physiologists at that time, that human intelligence was essentially different from the instinct of the invertebrates: "(...) every intellectual act is a product of an intimate movement of nervous substance whether it goes on in the human brain or in the ganglionic system of insects (...)." Even though Castro did not explicitly quote Darwin, that affirmation bears close resemblance to one notorious excerpt from the 1871 "The Descent of Man": "The difference in mind between man and the higher animals, great as it is, certainly is one of degree and not of kind".^9^ Regarding the frontal lobe functions, Castro acknowledges the seminal works on language by Paul Broca,^10^ and then turns to the subject of attention and its liaison to the motor centers. Likewise, he cites writings by the philosopher Alexander Bain and the psychologist Wilhelm Wundt on that matter. It is also worthy to compare that if Broca is the icon of cortical localization theory on clinical grounds, the German neurophysiologists Gustav Fritsch and Eduard Hitzig were the ones who first presented evidence for that theory in the laboratory, when they discovered a motor area in the cerebral cortex of dogs.^11^^,^ 12 Even though Castro did not refer them in his essay, he was probably familiar with their work because he had proficiency in the German language and the German school of medicine was highly influent in his writings.^1^ He may have chosen to address only Ferrier's experiments due to a larger similarity of his research subjects to humans.

It is important to mention Nuno de Andrade, a prominent physician who was partially responsible for the implementation of the psychiatry chair in Brazilian medical schools.^13^ In his critique of Castro's essay, Andrade refer to the works of Fritsch and Hitzig showing that he was up-to-date on the matter. However, Andrade was cautious not to fully embrace the doctrine of cortical localization, as some of the experiments seemed to him methodologically flawed. Furthermore, Andrade demonstrates a mature scientific mind on this subject, but nevertheless acclaims Castro in the last lines of his appraisal: "(...) none of the experiments employed is unquestionable. The deductions on which the physiologists arrived do not authorize us to emit a categorical affirmation. (...) I do not accept nor deny it. I belong to the group that waits and I am always ready to applaud all the efforts and to praise all the cultured and promising intelligences, like that of doctor Francisco de Castro".^4^

In short, the life of this outstanding person was brief but plentiful, and the death of the "divine master", due to a pneumonia he acquired after examining a patient, stunned his friends and pupils.^1^
